# Fasting glucagon as an independent risk indicator for CAD in patient with type 2 diabetes

**DOI:** 10.3389/fendo.2025.1749418

**Published:** 2025-12-11

**Authors:** Linlin Kong, Lina Chang, Jiamin Nie, Yian Gu, Xin Wang, Siyu Yan, Wantong Han, Hequn Sang, Shaofang Tang, Ming Liu, Qing He

**Affiliations:** Department of Endocrinology and Metabolism, Tianjin Medical University General Hospital, Tianjin, China

**Keywords:** cardiometabolic risk, complication, coronary artery disease, glucagon, type 2 diabetes mellitus

## Abstract

**Objective:**

To investigate the association between fasting glucagon levels and coronary artery disease (CAD) risk in patients with Type 2 Diabetes Mellitus (T2DM).

**Methods:**

This cross-sectional study enrolled 1,739 hospitalized T2DM patients, categorized into T2DM alone and T2DM with CAD (T2DM&CAD) groups. Fasting glucagon levels and clinical characteristics were collected. Multivariable logistic regression models were used to assess this association, with progressive adjustment for confounders.

**Results:**

In female patients, fasting glucagon levels were significantly higher in the T2DM&CAD group than in the T2DM alone group (13.33 vs. 11.52 pmol/L, P < 0.01). After full adjustment, each 1-SD increase was associated with a 49.2% higher CAD risk (OR: 1.492; 95% CI: 1.099-2.026; P < 0.05). No significant association was found in male patients.

**Conclusion:**

Elevated fasting glucagon is an independent risk indicator for CAD in women with T2DM, but not in men. These findings highlight the potential value of glucagon monitoring in T2DM management, especially for women, and support exploring glucagon-pathway-targeted therapies to reduce cardiovascular complications.

## 1 Introduction

Type 2 diabetes mellitus (T2DM) has become a major global public health problem, with its prevalence continuing to rise, placing a heavy burden on society (1). It is noteworthy that the incidence of cardiovascular disease (CVD) is significantly higher in diabetic populations compared to non-diabetic populations (2), and coronary artery disease (CAD) is a leading cause of death in T2DM patients, accounting for approximately 50% of mortality (3). This severe situation urgently requires in-depth exploration of the pathological mechanisms linking T2DM and CAD.

Current understanding of the comorbidity mechanisms of these two diseases primarily focuses on two aspects: first, traditional cardiovascular risk factors such as age, gender, smoking, and hypertension (4, 5); second, pathophysiological alterations specific to T2DM. T2DM is often accompanied by chronic low-grade inflammation and oxidative stress, processes that accelerate atherosclerosis through various pathways including endothelial dysfunction, macrophage infiltration and adhesion, and smooth muscle cell proliferation (6). Research indicates that insulin resistance is not only a characteristic of T2DM but also an independent predictor of coronary atherosclerotic plaque progression (7). Furthermore, T2DM patients exhibit enhanced platelet activity and dysregulation of coagulation factors, collectively contributing to a prothrombotic state (8). In recent years, researchers have identified some novel biomarkers, such as elevated C-peptide levels being independently associated with CAD risk in T2DM patients (9). However, these mechanisms still cannot fully explain the high incidence of cardiovascular disease in diabetic patients, suggesting the need to seek answers from new perspectives, such as hormonal imbalance.

Glucagon, as a key counter-regulatory hormone to insulin, is secreted by pancreatic alpha cells and maintains metabolic homeostasis by acting on multiple organs including the liver, kidneys, and heart (10). Of particular note, previous studies have indicated that increased glucagon levels are associated with an elevated risk of diabetic kidney disease (11). This finding prompts us to consider whether glucagon also participates in the pathogenesis of macrovascular complications, such as CAD, in T2DM patients. However, clinical research evidence on the relationship between glucagon and T2DM complicated by CAD is currently lacking. Therefore, this study, based on a retrospective analysis of clinical data, aims to investigate the relationship between fasting glucagon levels and the risk of CAD in T2DM patients, providing new insights for the early warning and precise intervention of diabetic cardiovascular complications.

## 2 Materials and methods

### 2.1 Subjects and study design

This study initially included a total of 2360 patients with type 2 diabetes mellitus who were hospitalized at Tianjin Medical University General Hospital from September 1, 2022, to September 30, 2025, and whose fasting glucagon was measured. According to the exclusion criteria, 621 cases were excluded, resulting in 1739 patients finally included in the study. Diagnostic Criteria: Diabetes was defined as fasting plasma glucose ≥7.0 mmol/L, 2-hour plasma glucose during an oral glucose tolerance test ≥11.1 mmol/L, random plasma glucose ≥11.1 mmol/L, glycosylated hemoglobin (HbA1c) ≥6.5%, self-reported history of diabetes, or use of hypoglycemic agents (12).The diagnosis of CAD was based on clinical presentation, electrocardiogram, coronary computed tomography angiography (CCTA), and invasive coronary angiography, in accordance with the 2024 ESC Guidelines for the management of chronic coronary syndromes (13).Dyslipidemia was defined as total cholesterol (TC) ≥ 6.2 mmol/L, triglycerides (TG) ≥ 2.3 mmol/L, low-density lipoprotein cholesterol (LDL-C) ≥ 4.1 mmol/L, or high-density lipoprotein cholesterol (HDL-C) < 1.0 mmol/L, or being on lipid-lowering medication (14). Exclusion Criteria: 1.Age <18 years or pregnancy status; 2. Administration of exogenous glucocorticoids; 3.Severe hepatic or renal insufficiency, gastrointestinal diseases, anemia, malignancy; 4.Comorbidity with other endocrine diseases affecting blood glucose levels, such as hyperthyroidism; 5. patients with any documented use of SGLT2 inhibitors (including current use or use at any time in the past) prior to admission or during the hospitalization period were excluded; 6.Missing data. Ethics: This study was approved by the Ethics Institutional Review Board of Tianjin Medical University General Hospital (IRB2020-YX-027-01).

### 2.2 Data collection

Hospitalization information of T2DM patients was collected from the electronic medical record system, including demographic characteristics, lifestyle, medical history, anthropometric indicators, laboratory results, and medication information. Demographic characteristics included: hospitalization number, gender, date of birth; lifestyle information included: smoking history, alcohol consumption history; medical history information included: hypertension, coronary artery disease, hyperuricemia, fatty liver, dyslipidemia; anthropometric indicators included: height, weight, systolic blood pressure (SBP), diastolic blood pressure (DBP); laboratory results included: fasting glucose, fasting C-peptide, fasting glucagon, total cholesterol (TC), triglyceride (TG), high density lipoprotein cholesterol(HDL-C),low density lipoprotein cholesterol(LDL-C), glycosylated hemoglobin(HbAlc), serum uric acid (SUA); medications Information includes: lipid-lowering drug, antihypertensive agents, Glucagon Like Peptide-1 receptor agonists, insulin, dipeptidyl peptidase 4 inhibitors.

### 2.3 Measurement of insulin, glucagon, and C-peptide

Plasma glucose was measured by the hexokinase method (Instrument model: HITACHI 008AS, Japan).

Insulin and C-peptide were measured by chemiluminescence immunoassay (Instrument model: ARCHITECT i2000, USA). Glucagon was measured by chemiluminescence immunoassay using a fully automated chemiluminescence analyzer (HomoG 100, China) employing dual monoclonal antibodies targeting the N- and C-termini of glucagon.

### 2.4 Statistical methods and related formulas

Continuous variables that conformed to a normal distribution were expressed as mean ± standard deviation, and the student-t test was used to compare the differences between the two groups. Continuous variables that are not normally distributed are expressed as median (upper and lower quartiles), and the Kruskal-Wallis rank sum test was used to compare the differences between the two groups. Categorical variables were expressed as numbers (frequencies), and the chi-square test was used to compare differences in distribution between the two groups. Binary logistic regression was used for multifactorial analysis, with CAD as the dependent variable and stepwise adjustment for covariates in the three models. SPSS 27.0 (Chicago, IL, USA) was used for all statistical analyses; a two-tailed P value <0.05 was considered statistically significant. The 2021 CKD-EPI eGFRcr was used (24).

## 3 Results

### 3.1 Clinical characteristics of patients with T2DM and T2DM&CAD

**Table 1** presents the clinical characteristics of patients with T2DM and T2DM complicated by CAD, stratified by gender. A total of 1739 patients were included. The baseline characteristics table of the T2DM with CAD group and the T2DM without CAD group is shown in **Supplementary Table 1**.

**Table 1:** Clinical characteristics of patients with T2DM and T2DM&CAD, grouped according to gender.

Characteristic	Female		Male	
	T2DM	T2DM&CAD	T2DM	T2DM&CAD
Number, n(%)	513	168	799	259
Age, year	60 (50, 68)	69 (62, 74)^***^a	54 (42, 64)	65 (57, 70)^***^b
T2DM duration, month	84 (12, 192)	180 (85.5)^***^a	69 (12, 144)	132 (60, 240)^***^b
Smoking, n(%)	28 (5.5)	14 (8.3)	384 (48.1)	122 (47.1)
Drinking, n(%)	12 (2.3)	2 (1.2)	353 (44.2)	96 (37.1)
Hypertension, n(%)	267 (52.0)	138 (82.1)^***^a	438 (54.8)	196 (75.7)^***^b
Fatty liver, n(%)	367 (71.5)	104 (61.9)^**^a	575 (72.0)	168 (64.9)^*^b
Dyslipidemia, n(%)	351 (68.4)	142 (84.5)^***^a	641 (80.2)	234 (90.3)^**^b
Hyperuricemia, n(%)	113 (22.0)	29 (17.3)	243 (30.4)	67 (25.9)
Lipid-lowering drug, n(%)	140 (27.3)	99 (58.9)^***^a	231 (28.9)	160 (61.8)^***^b
Antihypertensive agents, n(%)	213 (41.5)	117 (69.6)^***^a	324 (40.6)	163 (62.9)^***^b
DPP-4i, n(%)	128 (25.0)	44 (26.2)	171 (21.4)	59 (22.8)
GLP-1RA, n(%)	44 (8.6)	23 (13.7)	107 (13.4)	45 (17.4)
Insulin, n(%)	280 (54.6)	93 (55.4)	381 (47.7)	140 (54.1)
BMI, Kg/m2	25.39 (22.88, 28.50)	25.12 (23.05, 27.45)	26.81 (24.25, 29.91)	25.56 (23.39, 27.78)^***^b
BP, mmHg				
SBP	130 (121, 43.5)	134 (123, 149)^*^a	132 (121, 1430	133 (121, 147)
DBP	80 (74, 87)	78 (72, 83)^**^a	83 (77, 92)	80 (75, 89)^**^b
TC, mmol/L	5.06 (5.34, 5.91)	4.24 (3.58, 5.09)^***^a	4.80 (4.15 5.56)	4.06 (3.18, 4.87)^***^b
TG, mmol/L	1.69 (1.23, 2.45)	1.74 (1.22, 2.13)	1.90 (1.34, 2.96)	1.70 (1.16, 2.69)^**^b
HDL-C, mmol/L	1.04 (0.88, 1.20)	1.03 (0.88,.20)	0.91 (.80, 1.08)	0.89 (0.78, 1.04)
LDL-C, mmol/L	2.91 (2.35, 3.51)	2.22 (1.73, 2.9)^***^a	2.72 (2.21, 3.28)	2.09 (1.53, 2.75)^***^b
SUA, umol/L	308 (251, 378)	282 (242, 357)	357 (296, 435)	333.50 (288, 407)^*^b
eGFR, mL/min/1.73 m^2^	102.71 (95.23, 2.54)	94.45 (81.58, 101.04)^***^a	105.24 (94.91, 116.05)	98.57 (85.59, 104.74)^***^b
HbA1c, %	8.2 (7.2, 10.1)	7.7 (6.7, 9.43)^**^a	8.3 (6.9, 9.8)	8.0 (6.9, 9.1)^*^b

Data are presented as mean ± SD, median (Q1, Q4), or number (%).

Bold values indicate statistical significance, significance levels: *P<0.05, **P<0.01, ***P<0.001.

a, compared among female T2DM and T2DM complicated by CAD.

b, compared among male T2DM and T2DM complicated by CAD.

T2DM, type 2 diabetes mellitus; CAD, coronary artery disease; T2DM&CAD, type 2 diabetes mellitus complicated by coronary artery disease; DPP-4i, dipeptidyl peptidase 4 inhibitors;GLP-1RA, Glucagon Like Peptide-1 receptor agonists; BMI, body mass index; BP, blood pressure; SBP, systolic blood pressure; DBP, diastolic blood pressure; TC, total cholesterol; TG, triglyceride; HDL-C, high density lipoprotein cholesterol; LDL-C, low density lipoprotein cholesterol; SUA, serum uric acid; HbAlc, glycosylated hemoglobin.

In Females, compared to the T2DM group, the T2DM&CAD group was older (60 years vs. 69 years, P < 0.001), had a longer T2DM duration (84 months vs. 180 months, P < 0.001), and a higher prevalence of hypertension (52.0% vs. 82.1%, P < 0.001). The lipid-lowering medications, Dyslipidemia and antihypertensive drugs were also more frequent in the T2DM&CAD group (P < 0.05 for all). However, the prevalence of fatty liver disease was lower in the T2DM&CAD group (71.5% vs. 61.9%, P < 0.05), and eGFR, DBP, TC, LDL-C, HbA1c were lower (P < 0.05 for all).

In Males, The T2DM&CAD group was older (54 years vs. 65 years, P < 0.001), had a longer T2DM duration (69 months vs. 132 months, P < 0.001), and a higher prevalence of hypertension (54.8% vs. 75.7%, P < 0.001). The use of lipid-lowering and antihypertensive medications was also more frequent in the T2DM&CAD group (P < 0.05 for both). Additionally, the prevalence of Dyslipidemia was higher in the T2DM&CAD group (80.2% vs. 90.3%, P < 0.01), and eGFR, BMI, DBP, TC, LDL-C, HbA1c, and SUA were lower (P < 0.05 for all).

Notably, despite a higher prevalence of hypertension in the T2DM&CAD group, patients in this group exhibited lower lipid and blood pressure levels compared to those in the T2DM group. This discrepancy may be attributed to more aggressive lifestyle interventions (e.g., dietary control and exercise), higher use of lipid-lowering medications, and differences in antidiabetic drug regimens in the T2DM&CAD group. These findings highlight the importance of comprehensive management strategies in patients with T2DM complicated by CAD.

### 3.2 Analysis of differences in fasting glucagon, blood glucose, c-peptide, and insulin.

For female, fasting glucagon levels (13.33 vs.11.52, P < 0.01) and insulin (12.20 vs.17.40, P < 0.05) levels were higher in the T2DM&CAD group, while fasting blood glucose levels (6.65 vs.7.8, P < 0.001) were lower in the T2DM&CAD group. For male, no significant differences were found among T2DM and T2DM&CAD group, **Table 2**.

**Table 2:** Analysis of differences in fasting glucagon, blood glucose, c-peptide, and insulin.

Group	T2DM	T2DM&CAD	P
Female			
GCG0, pmol/L	11.52 (8.73, 16.17)	**13.33 (9.22, 18.42)**	**<0.01**
FBG, mmol/L	7.80 (6.10, 10.60)	**6.65 (5.50, 8.68)**	**<0.001**
FC-P, ng/mL	1.82 (1.08, 2.79)	1.99 (1.11, 2.93)	0.281
FINS, mU/L	12.20 (8.10, 20.30)	**17.40 (8.88, 31.50)**	**<0.05**
Male			
GCG0, pmol/L	14.93 (10.81, 21.99)	15.56 (10.81, 22.58)	0.780
FBG, mmol/L	7.50 (6.10, 9.90)	**7.00 (5.60, 8.70)**	**<0.01**
FC-P, ng/mL	1.88 (1.22, 2.94)	1.79 (1.15, 3.07)	0.392
FINS, mU/L	11.50 (7.20, 18.33)	12.05 (7.05, 24.88)	0.376

Data are presented as median (Q1, Q4).

T2DM, type 2 diabetes mellitus; CAD, coronary artery disease; T2DM&CAD, type 2 diabetes mellitus complicated by coronary artery disease; GCG0, fasting glucagon; FBG, fasting blood glucose; FC-P, fasting C-peptide; FINS, fasting insulin.

The number of patients (n) in each subgroup is consistent with **Table 1**; T2DM group (Female), n=513; T2DM&CAD group (Female), n=168; T2DM group (Male), n=799; T2DM&CAD group (Male), n=259.

Bold values indicate statistical significance.

### 3.3 Fasting glucagon analysis in T2DM and T2DM&CAD

**Supplementary Table 2** provides the VIF values for each covariate. In three multivariable logistic regression models, we progressively adjusted for confounding factors and examined the relationship between fasting glucagon levels and the risk of T2DM complicated by CAD separately in females and males, **Table 3**. For female patients, Model 1 was unadjusted; Model 2 was adjusted for age, smoking history, alcohol use, and T2DM duration based on Model 1; and Model 3 was further adjusted for BMI, fatty liver, hypertension, eGFR, fasting C-peptide, HbA1c, Dyslipidemia, serum uric acid, Antihypertensive agents, use of GLP-1RA, use of DPP-4i, and use of insulin based on Model 2. Elevated plasma glucagon levels were significantly associated with an increased risk of CAD in all models (Model 1: OR, 1.038; 95% CI, 1.009-1.069; P < 0.05; Model 2: OR, 1.046; 95% CI, 1.013-1.080; P < 0.01; Model 3: OR, 1.049; 95% CI, 1.011-1.087; P < 0.05). When glucagon was included as a per standard deviation (SD) increase in Models 1, 2, and 3, each 1-SD increase in fasting glucagon was significantly associated with a 37.1%, 46.2%, and 49.2% higher risk of CAD, respectively (Model 1: OR, 1.371; 95% CI, 1.074-1.750; P < 0.05; Model 2: OR, 1.462; 95% CI, 1.118-1.912; P < 0.01; Model 3: OR, 1.492; 95% CI, 1.099-2.026; P < 0.05). In contrast, fasting glucagon levels were not significantly associated with the risk of T2DM complicated by CAD in males (all P > 0.05).

**Table 3:** Binary logistic regression analysis of fasting glucagon in patients with T2DM and T2DM&CAD.

Group		OR (95%CI)	
	Model 1	Model 2	Model 3
Female			
GCG, pmol/L	1.038 (1.009, 1.069)^*^	1.046 (1.013, 1.080)^**^	1.049 (1.011, 1.087)^*^
Per-SD increase	1.371 (1.074, 1.750)^*^	1.462 (1.118, 1.912)^**^	1.492 (1.099, 2.026)^*^
Male			
GCG, pmol/L	1.004 (0.986, 1.022)	1.016 (0.996, 1.036)	1.011 (0.989, 1.033)
Per-SD increase	1.034 (0.886, 1.206)	1.141 (0.963, 1.352)	1.099 (0.915, 1.321)

Bold values indicate statistical significance, significance levels: *P<0.05, **P<0.01.

Model 1, No adjustment was made.

Model 2, Adjusted for age, smoking history, alcohol consumption history and T2DM disease duration based on model 1.

Model 3, Adjusted for BMI, fatty liver, hypertension, eGFR, fasting C-peptide, HbA1c, Dyslipidemia, serum uric acid, Antihypertensive agents, use of GLP-1RA, use of DPP-4i, and use of insulin based on model 2.

## 4 Discussion

This study, by analyzing data from 1,739 hospitalized T2DM patients, identified for the first time an independent association between fasting glucagon levels and CAD risk specifically in the female cohort. This finding provides a new perspective for understanding the pathogenesis of cardiovascular complications in diabetes. After adjusting for multiple confounding factors including age, disease duration, lifestyle, metabolic parameters, and medication use, each standard deviation increase in fasting glucagon was associated with a 49.2% increased risk of CAD in female T2DM patients. This significant dose-response relationship suggests that glucagon may play an important role in the development and progression of CAD in women with T2DM.

Glucagon may promote CAD development through several pathways. Firstly, by binding to hepatic GCGR and activating the cAMP-PKA pathway, glucagon simultaneously promotes glycogenolysis and gluconeogenesis, exacerbating the hyperglycemic state (15–18). Persistent hyperglycemia can promote oxidative stress and inflammatory responses through various pathways including the polyol pathway, hexosamine pathway, AGEs accumulation, PKC activation, and NF-κB signaling, thereby accelerating the process of vascular pathology (8). The female CAD group in our study indeed demonstrated higher glucagon levels, supporting this hypothesis.

Secondly, glucagon may form a vicious cycle with insulin resistance. Animal experiments have shown that inhibiting GCGR signaling improves branched-chain amino acid metabolism, enhances cardiac function after myocardial infarction, and attenuates cardiac remodeling by modulating the TAK1/p38 MAPK and KLF15/BCATm pathways (19). Clinical studies have also found that antagonizing glucagon signaling not only improves glycemic control but also enhances insulin sensitivity (20). This suggests that elevated glucagon may indirectly increase CAD risk by worsening insulin resistance.

In addition to these indirect mechanisms, the direct effects of glucagon on the heart cannot be ignored. The expression of GCGR in cardiac tissue has been confirmed (21–23), but its effects appear to be context-dependent. Pharmacological doses of glucagon produce positive inotropic effects and can be used to treat overdose of beta-blockers or calcium channel blockers (24); however, in ischemic hearts, glucagon may exacerbate myocardial injury through a p38 MAPK-dependent pathway. Correspondingly, mice with cardiomyocyte-specific knockout of GCGR showed better survival and less cardiac remodeling after myocardial infarction (25). Another study demonstrated that the anti-GCGR antibody REMD 2.59 significantly improved cardiac function and tissue remodeling in mice with myocardial infarction (26). These seemingly contradictory results suggest that the cardiac effects of glucagon may be highly dependent on the body’s metabolic state, which will be an important direction for future research.

Mendelian randomization studies in the general population suggest that genetically determined higher glucagon levels may be a potential risk factor for ischemic heart disease (27). This provides genetic evidence for the cardiovascular effects of glucagon beyond traditional risk factors. On the other hand, within the field of diabetes, previous studies have begun to focus on the association between glucagon and microvascular complications. One cross-sectional study indicated that, after adjusting for covariates including demographic characteristics, lipid-lowering drugs, and hypoglycemic agents, increased glucagon levels were independently associated with an increased risk of diabetic kidney disease (11). This forms a useful logical extension to our study: the potential pathological role of glucagon may involve both microvascular and macrovascular complications.

The sex heterogeneity found in this study may be related to the unique fluctuations in estrogen levels in women: the vasoprotective effects of premenopausal estrogen may partially counteract the detrimental effects of glucagon, while the decline in estrogen levels after menopause may weaken this protective effect (28). From a clinical practice perspective, our findings suggest that for female T2DM patients, fasting glucagon levels might have the potential to serve as an auxiliary biomarker to help identify individuals at higher risk for coronary heart disease. Building upon traditional risk factors (such as hypertension and dyslipidemia), incorporating glucagon level assessment might enable more precise risk stratification. Secondly, the research results provide a theoretical basis for the development of glucose-lowering drugs targeting the glucagon pathway. GLP-1 receptor agonists, while effectively lowering blood glucose, also suppress glucagon secretion, and their cardiovascular protective effects have been confirmed in several clinical trials (29). Dual GLP-1/GCGR agonists and triple GLP-1/GIP/GCGR agonists (30) currently under development have shown significant advantages in controlling body weight and blood glucose. Whether they can provide additional benefits for T2DM patients with CAD warrants further investigation.

This study has several limitations that should be considered when interpreting the results. First, and most crucially, the cross-sectional design prevents us from inferring causality. Second, although we made considerable efforts to adjust for numerous confounding factors, particularly correcting for medications affecting glucagon, the possibility of residual confounding cannot be completely ruled out. Furthermore, the study participants were inpatients from a single tertiary hospital, potentially limiting the generalizability of the population and introducing selection bias. As all participants were inpatients from a tertiary care center, our sample likely represents a higher-risk population, which may limit generalizability to community-based or primary care settings. The applicability of the findings to community populations or other ethnic groups requires further validation. Also, CAD severity (e.g., number of affected vessels, prior MI, or CCTA scores) was not systematically graded, which may have attenuated potential dose–response relationships between glucagon levels and coronary atherosclerotic burden.

## 5 Conclusion

This cross-sectional study found that fasting glucagon levels are independently associated with the risk of CAD in female patients with T2DM.These results suggest that glucagon may play an important role in the cardiovascular pathology of female T2DM patients and could be a potential novel biomarker for CAD risk. However, given the observational design, these findings are hypothesis-generating and provide a rationale for further investigation into the mechanisms of the glucagon pathway in diabetic cardiovascular complications and related intervention strategies.

## Data Availability

The raw data supporting the conclusions of this article will be made available by the authors, without undue reservation.
