# Supplementary material for: Fasting glucagon as an independent risk indicator for CAD in patient with type 2 diabetes
Source: Front Endocrinol (Lausanne). 2025 Dec 11;16:1749418. doi: 10.3389/fendo.2025.1749418 (PMC12738176; doi:10.3389/fendo.2025.1749418)
Supplement: Supplementary file 1 [file Table1.docx]

**Supplementary Table 1** Clinical characteristics of patients with T2DM and T2DM&CAD

|  | T2DM | | T2DM&CAD |
| --- | --- | --- | --- |
| Number，n（%） | 1312 | 427 | |
| Age，year | 57 (44, 66) | **66 (60. 73)^***^** | |
| T2DM duration，month | 72 (12, 168) | **156 (72, 240)^***^** | |
| Smoking，n（%） | 412 (31.4) | 136 (31.9) | |
| Drinking，n（%） | 365 (27.8) | 98 (23.0) | |
| Hypertension，n（%） | 705 (53.7) | **334 (78.2)^***^** | |
| Fatty liver，n（%） | 942 (71.8) | **272 (63.7)^**^** | |
| Dyslipidemia，n（%） | 992 (75.6) | **376 (88.1)^***^** | |
| Hyperuricemia，n（%） | 356 (27.1) | 96 (22.5) | |
| Lipid-lowering drug，n（%） | 371 (28.3) | **259(60.7)^***^** | |
| Antihypertensive agents，n（%） | 537 (40.9) | **280 (65.6)^***^** | |
| DPP-4i，n（%） | 299 (22.8) | 103 (24.1) | |
| GLP-1RA，n（%） | 151 (11.5) | 68 (5.9) | |
| Insulin，n（%） | 661 (50.4) | 233 (54.6) | |
| BMI，Kg/m2 | 26.28 (23.66, 29.32) | **25.39 (23.34, 27.78)^**^** | |
| BP，mmHg |  |  | |
| SBP | 131 (121, 143) | 133 (122, 148) | |
| DBP | 82 (75, 90) | **79 (74, 87)^***^** | |
| TC，mmol/L | 4.90 (4.20, 5.71) | **4.10 (3.33, 4.97)^***^** | |
| TG，mmol/L | 1.80 (1.30, 2.73) | **1.70 (1.18, 2.45)^**^** | |
| HDL-C，mmol/L | 0.97 (0.82, 1.13) | 0.94 (0.82, 1.10) | |
| LDL-C，mmol/L | 2.78 (2.25, 3.36) | **2.14 (1.58, 2.81)^***^** | |
| SUA，umol/L | 337.50 (274.00, 412.25) | **320.00 (263.00, 390.00)^*^** | |
| eGFR, mL/min/1.73 m^2^ | 104.43 (95.09, 114.63) | **96.60 (84.77, 103.66)^***^** | |
| HbA1c，% | 8.3 (7.0, 9.9) | **7.8 (6.9, 9.2)^***^** | |

Data are presented as mean ± SD, median (Q1, Q4), or number (%).

Significance levels：*P<0.05，**P<0.01，***P<0.001.

T2DM: type 2 diabetes mellitus; CAD: coronary artery disease; T2DM&CAD: type 2 diabetes mellitus complicated by coronary artery disease; DPP-4i: dipeptidyl peptidase 4 inhibitors;GLP-1RA: Glucagon Like Peptide-1 receptor agonists; BMI: body mass index; BP: blood pressure; SBP: systolic blood pressure; DBP: diastolic blood pressure; TC: total cholesterol; TG: triglyceride; HDL-C: high density lipoprotein cholesterol; LDL-C: low density lipoprotein cholesterol; SUA: serum uric acid; HbAlc: glycosylated hemoglobin.

**Supplementary Table 2** VIF of patients with T2DM and T2DM&CAD

|  | VIF | |
| --- | --- | --- |
|  | Female | Male |
| Age，year | 2.34 | 2.03 |
| T2DM duration，month | 1.04 | 1.03 |
| Smoking，n（%） | 1.09 | 1.25 |
| Drinking，n（%） | 1.09 | 1.24 |
| Hypertension，n（%） | 2.23 | 1.93 |
| Fatty liver，n（%） | 1.32 | 1.27 |
| Dyslipidemia，n（%） | 1.13 | 1.13 |
| Antihypertensive agents，n（%） | 2.17 | 1.93 |
| DPP-4i，n（%） | 1.08 | 1.07 |
| GLP-1RA，n（%） | 1.09 | 1.12 |
| Insulin，n（%） | 1.36 | 1.39 |
| BMI，Kg/m2 | 1.53 | 1.09 |
| SUA，umol/L | 1.39 | 1.41 |
| eGFR, mL/min/1.73 m^2^ | 2.27 | 1.93 |
| HbA1c，% | 1.39 | 1.28 |
| FC-P，ng/mL | 1.47 | 1.55 |

DPP-4i: dipeptidyl peptidase 4 inhibitors;GLP-1RA: Glucagon Like Peptide-1 receptor agonists; BMI: body mass index; SUA: serum uric acid; HbAlc: glycosylated hemoglobin; FC-P: fasting C-peptide.
